# Study on the Effect of Regeneration Agent on the Viscosity Properties of Aged Asphalt

**DOI:** 10.3390/ma15010380

**Published:** 2022-01-05

**Authors:** Jiangang Yang, Luhua Luo, Jie Gao, Jing Xu, Chengping He

**Affiliations:** 1 School of Civil Engineering and Architecture, East China Jiaotong University, Nanchang 330013, China; jekong@ecjtu.edu.cn (J.Y.); jingxv@ecjtu.edu.cn (J.X.); 2 Institute of Road Engineering, East China Jiaotong University, Nanchang 330013, China; 3 School of Transportation and Logistics, East China Jiaotong University, Nanchang 330013, China; luoluhua@163.com; 4 Fujian Expressway Maintenance Engineering Co., Ltd., Fuzhou 350001, China; hechengping@163.com

**Keywords:** road engineering, viscosity properties, gel permeation chromatography, atomic force microscope, aged asphalt

## Abstract

China’s highway asphalt pavement has entered the stage of major repair, and improving the utilization rate of recycled asphalt pavement (RAP) is the main issue. The key link affecting the performance of recycled asphalt mixtures is the regeneration of aged asphalt, and the effect of the regenerant dosing on the high-temperature performance and viscosity of aged asphalt is the main content to be studied in this research. The aging behavior of asphalt seriously affects the roadworthiness of asphalt mixtures. In this study, we investigated the effect of changes in the microscopic properties of the aged asphalt on its viscosity properties during regeneration using gel permeation chromatography (GPC), Fourier-transform infrared spectroscopy (FTIR), and atomic force microscopy (AFM) as well as Brinell viscosity tests. This study simulated asphalt aging by the RTFOT test, and then we obtained an aged asphalt with a needle penetration of 30. We prepared different regenerated asphalts by adding regeneration agent with doses of 2%, 4%, and 6% to the aged asphalt. The results showed that the regeneration agent could effectively reduce the viscosity of the aged asphalt, which can play a positive role in improving the construction and ease of the aged asphalt. Rejuvenation agents affected the aging asphalt sulfoxide and carbon group indices. Moreover, rejuvenation agents can also significantly reduce the intensities of their characteristic functional group indices. The results of the AFM test showed that the increase in the dose of regeneration agent increased the number of the asphalt bee-like structures and decreased the area of individual bee-like structures. The results of the GPC test were consistent with the results of the AFM test, and the increase in the dose of regeneration agent reduced the asphalt macromolecule content. The viscosity properties and microstructure of the aged asphalt changed positively after the addition of the regeneration agent, indicating that the regeneration agent had a degrading and diluting effect on macromolecules.

## 1. Introduction

Asphalt pavements are subject to deterioration by light, heat, and oxygen during service, resulting in the deterioration of pavement performance [1,2]. The critical factor in the regeneration of asphalt pavements is the regeneration of aged asphalt. Springback agents are generally used to restore the performance of aged asphalt during the reuse of asphalt pavements. The literature shows [3] that the main types of regeneration agents include vegetable oils, waste-derived oils, engineering products, and various refinery base oils. Previous studies on asphalt regeneration have focused more on the effects of regeneration agents on asphalt and the macroscopic properties of asphalt mixtures [4]. Still, in recent years, scholars at home and abroad have turned their attention to studying the mechanism of asphalt regeneration and have achieved specific results.

Huang et al. [5] explored the interaction between new asphalt, old asphalt, and regeneration agent by Fourier-transform infrared spectroscopy (FTIR) and gel permeation chromatography (GPC). Moreover, the results showed that the changes of the functional group index and the molecular weight distribution could explain the regeneration effect of the regeneration agent. The parameters of the sulfoxide index and the GPC of qiFTIR can be used as reliable indicators to predict the macroscopic properties of asphalt. Lin et al. [6] studied the changes of the characteristic functional group content of asphalt before and after regeneration using FTIR. The results showed that the addition of the regeneration agent made the characteristic functional group content of regenerated asphalt decrease. Zhao et al. [7] studied regeneration agent changes on asphalt’s relative molecular weight distribution using gel permeation chromatography. The results showed that the regeneration agent reduced the macromolecular content of the aged asphalt, which effectively reduced the viscosity of the asphalt. Cui et al. [8] used atomic force microscopy (AFM) tests to obtain Young’s modulus (DMT) and adhesion indexes and explored the changes of regeneration agents on asphalt viscoelastic properties. From the study results, we can see that the DMT of the regenerated asphalt was reduced after the addition of the recycler. As a result, the viscoelastic properties were well recovered, and the adhesion properties of the asphalt–aggregate interface were enhanced. Li et al. [9] analyzed the effect of regeneration agents on the microstructure of aged asphalt using FTIR and AFM. The results showed that the regeneration agents made the absorption intensity of some specific functional groups decrease, enhanced the asphalt molecular motility and decreased the asphalt viscosity. He et al. [10] used infrared spectroscopy to explore the mechanism of reagents on SBS-modified asphalt. The results showed that a layer of interfacial film was formed between asphaltene and a soft pitch due to the addition of reagents, which further played a role in lubricating the asphalt, thereby reducing the viscosity of the aging asphalt. Yao [11] studied the mechanism of the effect of regeneration agents on aged asphalt, and the results showed that the regeneration agent could regulate the molecular weight distribution of the aged asphalt.

In summary, the current research on asphalt regeneration mainly focuses on the mechanism of asphalt regeneration and the prediction of post-regeneration performance, and fewer studies combined microscopic morphology and macroscopic performance on the change law of the viscosity performance of aged asphalt after regeneration. In this study, indoor simulated aging tests were conducted on the matrix asphalt, from which aged asphalt with a needle penetration of 30 was obtained, and the doses of a springback agent were 2%, 4%, and 6%. GPC, FTIR, and AFM tests, as well as activation energy, were used to analyze the changes of the relative molecular weight, chemical components, microstructure, and viscosity properties of asphalt before and after regeneration and to explore the change law of the microscopic properties of the regenerated asphalt and its influence on the viscosity properties.

## 2. Experimental Methods

### 2.1. Raw Materials

**Figure 1:**
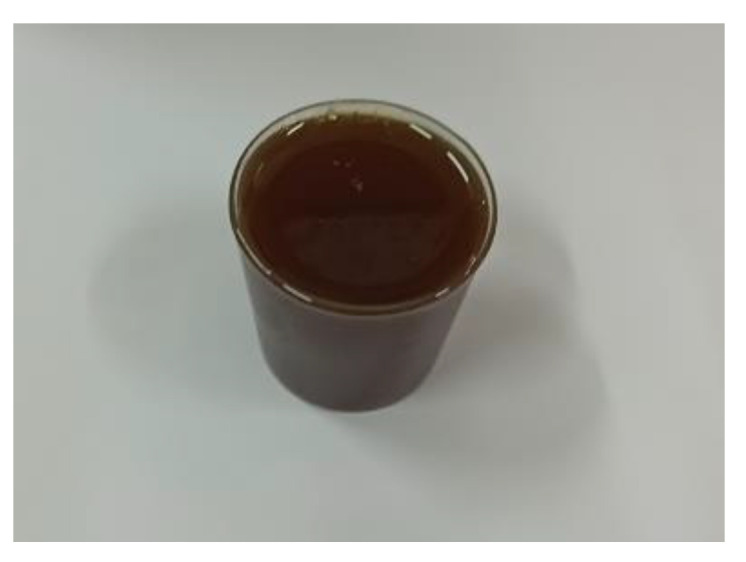
Regeneration agent.

Asphalt’s primary performance test results are shown in Table 1, and its main indexes met the specification requirements [12]. The test materials included 70# matrix asphalt and a regeneration agent. Figure 1 illustrates a sample of rejuvenators, and Table 2 describes specific technical properties.

### 2.2. Test method

#### 2.2.1. Aging Asphalt

**Figure 2:**
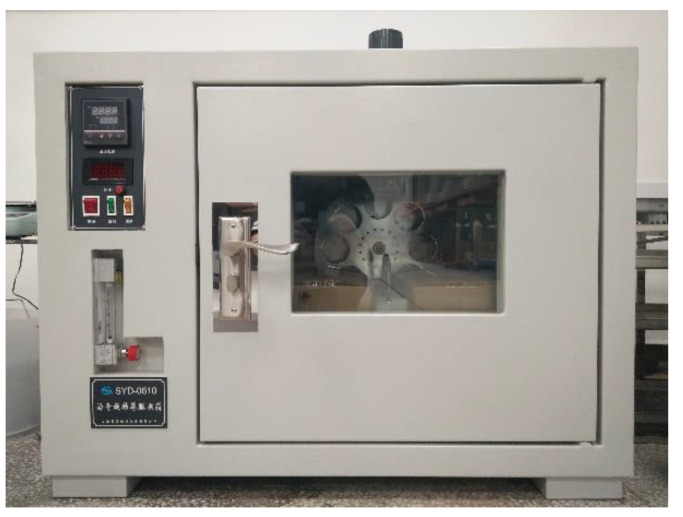
Rotating film oven.

**Figure 3:**
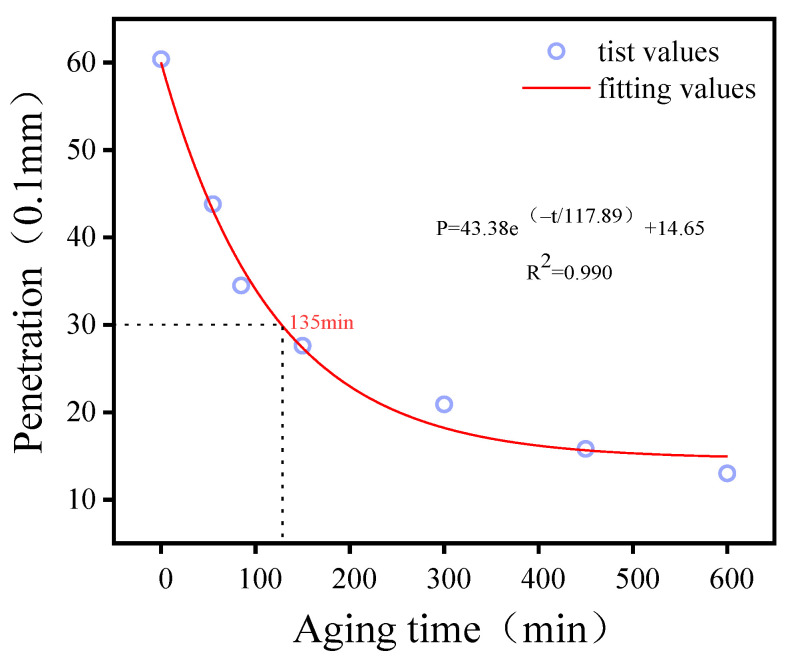
Needle penetration depths at different aging times in the RTFOT.

The rotating film oven (RTFOT; Figure 2) was used to age the 70# matrix asphalt into the asphalt with a needle penetration (0.1 mm) of 30. The aging time required for the asphalt was determined by relating the aging time to the degree of aging [13], and the results are shown in Figure 3. One hundred and thirty-five minutes were the aging time required for the 70# matrix asphalt to meet the performance index of 30# asphalt after aging.

#### 2.2.2. Asphalt Regeneration

The regeneration agent was similar to asphalt in chemical properties, and they had good compatibility. The aged asphalt was heated to the flowing state, and the regeneration agent with doses of 0%, 2%, 4%, and 6% were added and stirred for 30 min at 140 °C and 1000 rad/min using a high-speed shear to produce the regenerated asphalt. The preparation process referred to the literature [14]. For the convenience of presentation, the regenerated asphalts with the regeneration agent doping of 0%, 2%, 4%, and 6% was recorded as RA0, RA2, RA4, and RA6, respectively, and the 70# matrix asphalt was recorded as MA, as shown in Table 3.

#### 2.2.3. Physical Properties Test

JTG E20-2011 “Highway Engineering Asphalt and Asphalt Mixture Experimental Procedure” [15] was used to determine the physical properties of the regenerated asphalt, including the softening point (T0606-2011), the ductility at 15 °C (T0605-2011), and needle penetration at 25 °C (T0604-2011).

#### 2.2.4. Viscosity Test

A Brookfield viscometer with a temperature control device was selected to determine the viscosities of various asphalts. Due to the low viscosity of the target asphalt, we measured the viscosities of 120 °C, 135 °C, and 150 °C at 20 rad/min according to T0625-2011. To further analyze the change of the asphalt viscosity, we chose activation energy to analyze the change of the viscosity of the regenerated asphalt. Activation energy is the minimum energy required for molecules to reach the activation molecule to do work [16]. Activation energy can reflect the difficulty of the asphalt material to reach the flow state; the lower the activation energy, the better the construction and ease. The Arrhenius equation was described as:

(1) $$Ln\;\eta =\frac{E_{\eta }}{RT}+LnA$$

where *η* is the viscosity of asphalt (Pa·s); *T* is the absolute temperature (K); *A* is a constant; E*_η_* is the activation energy of asphalt when it undergoes change (kJ·mol^−1^); *R* is the universal gas constant with a magnitude of 8.314 J·mol^−1^ K^−1^. The study used the Brinell viscosity to derive the activation energy of the regenerated asphalt, which was utilized to analyze the construction and ease of regenerated asphalt [17].

#### 2.2.5. GPC Test

**Figure 4:**
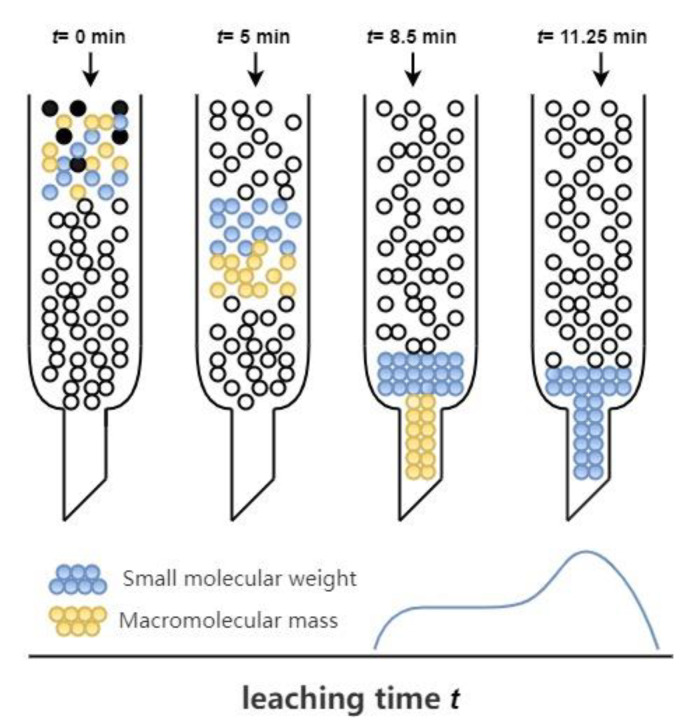
Gel permeation chromatography (GPC) schematic diagram.

An Agilent PL-GPC220 gel permeation chromatography was used to dissolve the asphalt specimens in a tetrahydrofuran (THF) solvent to form a solution at a specific concentration, with a specimen volume of 100 μL and a flow rate of 1 mL·mL^−1^. The column used to separate the asphalt specimens was maintained at 35 °C. The molecular weight and molecular weight size distribution of the asphalt were determined experimentally. Figure 4 shows the principle of GPC. In the GPC test, the separation mode was not based on the molecular weight, but molecules’ apparent size and molecular aggregation in a specific solution. After the asphalt sample was adequately dissolved, it was introduced into a group of columns through the injection mechanism as a molecular filtration system. The chromatographic column was filled with a cross-linked gel containing surface pores, which were different in size and played the role of molecular filters. Therefore, larger-size molecules (LMSs) were not able to enter smaller pores, and smaller molecules (MMSs) fit most pores and remained for a longer time.

GPC research results can show the differences of asphalt in molecular size distribution. In other words, GPC provided the “fingerprint” of each asphalt and provided a reasonable explanation for the changes of its macro-properties in combination with the knowledge of the relative molecular weight of asphalt components. Equations (2) and (3) show the formulae for the calculation of *M*_n_ (number average molecular weight) and *M*_w_ (heavy average molecular weight), respectively [18]:

(2) $$\overset{—}{M_{n}}=\frac{\sum H_{i}}{\sum (H_{i}/M_{i})},$$

(3) $$\overset{—}{M_{w}}=\frac{\sum M_{i}H_{i}}{\sum H_{i}},$$

where *H*_i_ is the peak height, *M*_i_ is the molecular weight, $\overset{—}{M_{n}}$ is the number average molecular weight, and $\overset{—}{M_{w}}$ is the heavy average molecular weight.

#### 2.2.6. FTIR Spectroscopy Test

FTIR can be used to detect chemical functional groups in solids, gases, and liquids. Many researchers have now used FTIR spectroscopy to characterize the aging and regeneration behavior of asphalt and the content of polymers [19]. In this study, a Breaker Alpha FTIR spectrometer with a spectral acquisition range of 500–4000 cm^−1^ was used to determine the functional groups of each asphalt specimen. OMNIC software was used for the baseline correction and smoothing of the IR curves to evaluate the regenerative effect of the regenerator on the aging asphalt with the use of the characteristic functional group (C=O and S=O) indices and the equation method [20] to calculate the sulfoxide group index (IS=O) and the carbonyl group index (IC=O) with the equations as in Equations (4) and (5):

(4) $$I_{S=O}=\frac{A_{1032}}{A_{2923}+A_{2852}},$$

(5) $$I_{C=O}=\frac{A_{1700}}{A_{2923}+A_{2852}}.$$

#### 2.2.7. AFM Test

**Figure 5:**
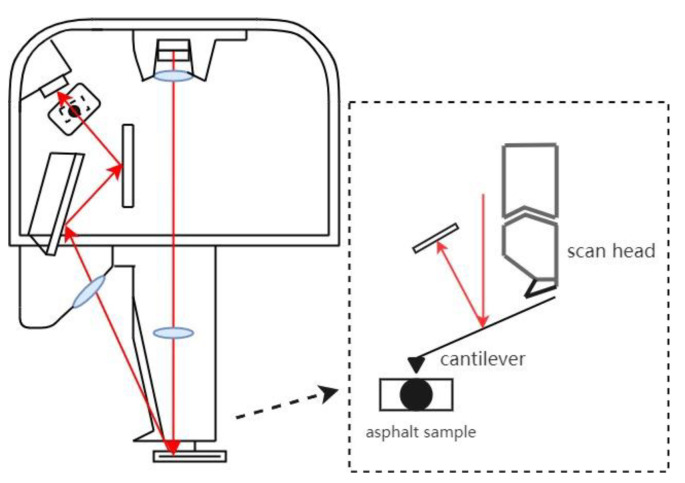
Schematic diagram of the atomic force microscopy (AFM) working principle.

**Figure 6:**
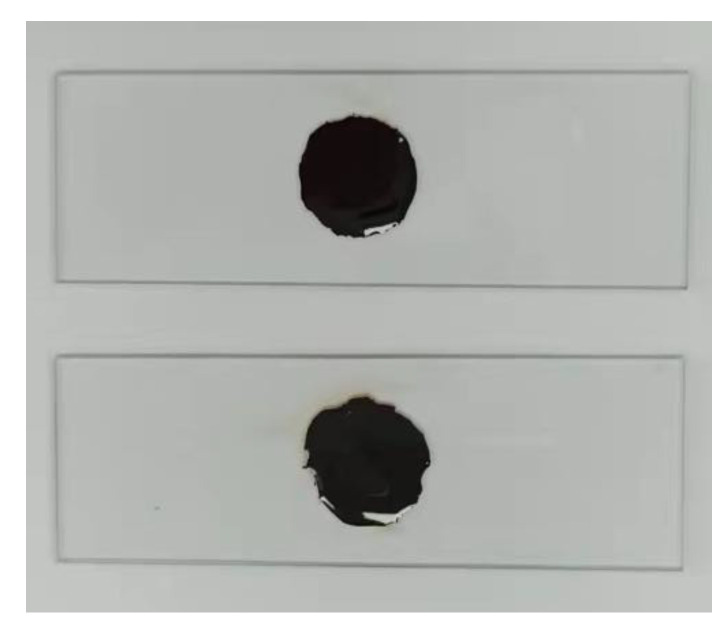
AFM specimens.

A Bruker Dimension Icon AFM was used to observe the surface morphologies of the asphalt specimens to obtain the asphalt morphology map. The test was performed in the tap mode with a scanning area of 20 μm × 20 μm. Figure 5 shows the schematic diagram of the AFM working principle. Figure 6 shows the asphalt AFM specimens, and the asphalt specimens were hot-cast. Firstly, the asphalt was heated to the flowing state, and then, a small amount of asphalt was dropped on the center of a slide and heated to make it flow freely on the slide. Finally, it was tested after natural cooling.

**Figure 7:**
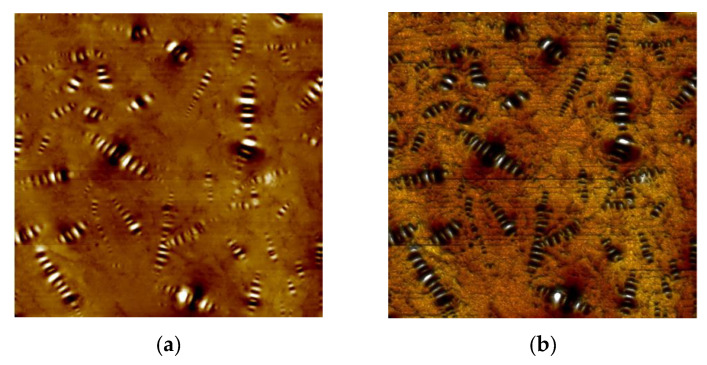

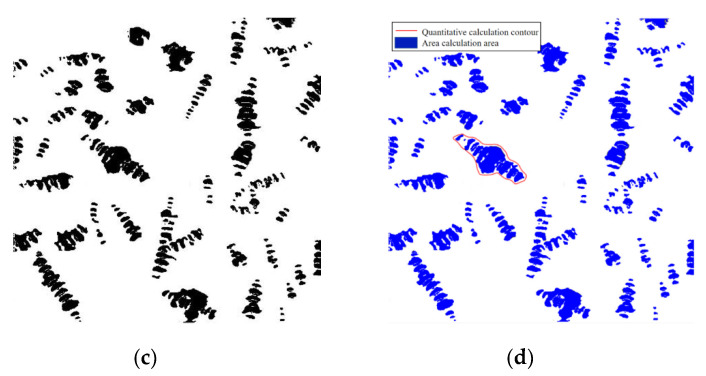
Quantitative analysis process of the asphalt micromorphological map processing: (**a**) original morphology; (**b**) pre-processing; (**c**) identification; (**d**) calculation.

*R_q_* and *R_a_* could characterize the roughness in the asphalt microscopic appearance and were described as Equations (6) and (7), respectively [21]:

(6) $$R_{q}=\sqrt{\frac{\sum_{1}^{N}Z_{i}^{2}}{N}},$$

(7) $$R_{a}=\frac{1}{N}\sum_{j=1}^{N}\left|Z_{j}\right|,$$

where *R_q_* is the average root mean square of the planar adhesion deviation, *R_a_* is the arithmetic mean of the absolute values of the adhesion deviation measured in the average plane, and *Z_i_* denotes the corresponding adhesion deviation. In order to further quantitatively investigate the microscopic property change of the regenerated asphalt, in this paper, the AFM morphological images were pre-processed and quantitatively calculated. Figure 7 shows the image processing and calculation process.

## 3. Analysis of Test Results

### 3.1. Analysis of Physical Properties Test Results

**Figure 8:**
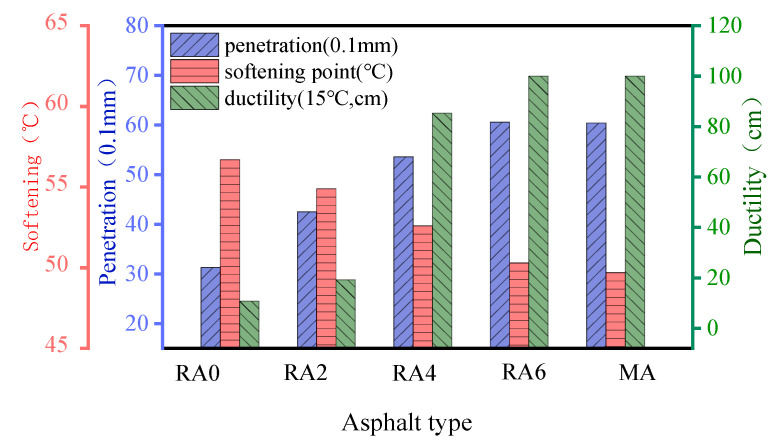
Physical properties of the regenerated asphalt at different doses.

As shown in Figure 8, the performance of the regenerated asphalt improved with the increase of the regenerator dose during the aging asphalt regeneration process. The needle penetration and the ductility increased significantly. In contrast, the softening point showed a decreasing trend. When the regenerator dose was 6%, the indicators of RA6 and MA tended to be close to each other, which indicated that the three primary indicators of the regenerated asphalt met the technical requirements of 70# base asphalt.

### 3.2. Viscosity and Activation Energy Analysis

**Figure 9:**
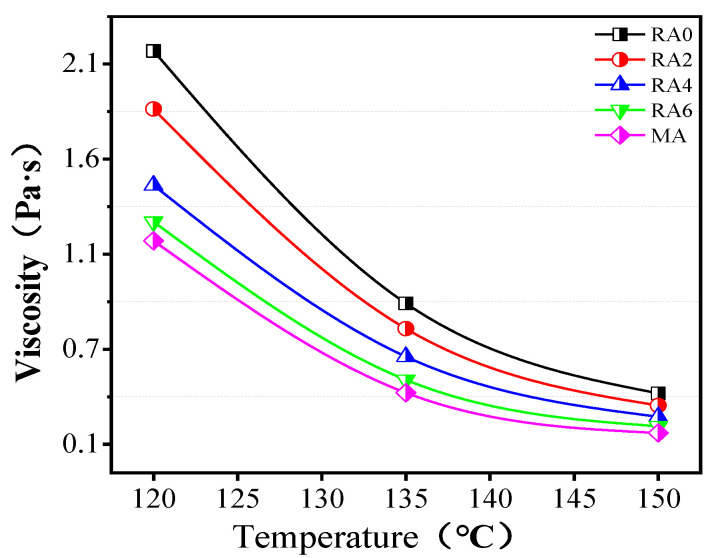
Viscosities of different asphalts.

**Figure 10:**
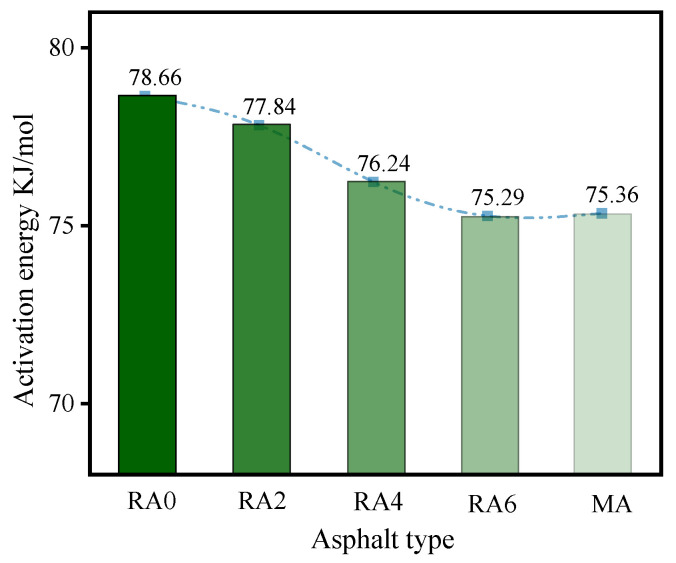
Activation energies of different asphalt viscosities.

The asphalt viscosity was closely related to the construction and ease. As seen in Figure 9, the asphalt viscosity tended to decrease, as the dosage of the regenerator increased. The viscosities difference between RA6 and MA was slight, indicating that the viscosity of the regenerated asphalt with a dose of 6% was close to the base asphalt. It indicated that the regenerator had the effect of reducing the viscosity of aging asphalt. The viscosity activation energy reflected the energy required for the regenerated asphalt to reach the flow state, in order to characterize the construction and ease of the regenerated asphalt. The smaller the viscosity activation energy, the better the construction and ease. Figure 10 shows that the viscosity activation energy of asphalt increased after aging. In addition, the activation energy of RA2, RA4, and RA6 decreased by 1.1%, 3.2%, and 4.6%, respectively, compared with that of RA0, indicating that the regeneration agent can reduce the viscosity of the aged asphalt and improve the construction. In addition, the activation energy of the viscosity of aged asphalt was similar to that of the base asphalt, when the dose of the rejuvenator was 6%.

### 3.3. Analysis of GPC Test Results

#### 3.3.1. Molecular Weight and Polydispersity

GPC provided valuable data about molecular weight distribution. Table 4 gives the GPC parameters of each asphalt specimen, including *M*_n_ (number average molecular weight), *M*_w_ (heavy average molecular weight), and polydispersity coefficient (PD = *M*_w_/*M*_n_). Generally, the larger the polydispersity index is, the wider the molecular weight distribution is. In addition, polydispersity represents the degree of component migration [22]. As shown in Table 4, the *M*_n_ value decreased with the increasing dose of the regenerator, which means that the asphalt formed smaller molecules through physical or chemical reactions during the regeneration process. By observing changes in the *M*_w_ and PD values of various asphalts, we can see that the *M*_w_ and PD of RA6 were lower than those of other asphalts, indicating that the regeneration agent led to lower *M*_w_ and PD values and had a degrading and diluting effect on the aged asphalt macromolecules.

#### 3.3.2. Molecular Weight Distribution

**Figure 11:**
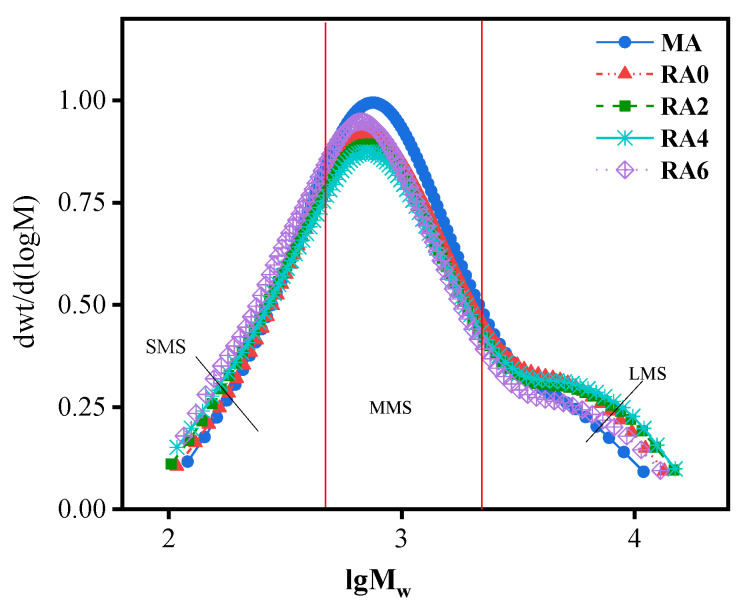
Chromatograms of the original asphalt and the regenerated asphalt.

**Figure 12:**
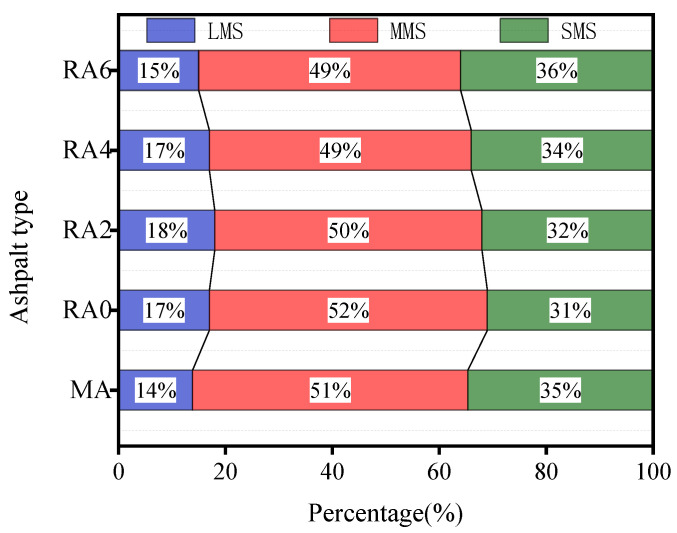
Molecular size distributions of the asphalt specimens.

Figure 11 shows the relative molecular mass (*M*_n_) distribution structure of the 70# matrix asphalt and the regenerated asphalt. The horizontal coordinate is the logarithm of the heavy average molecular weight, and the vertical coordinate is the relative content of asphalt. From Figure 11, we can see that the molecular weight of asphalt was mainly in the range of 10^2^–10^4^ and the molecular weight distribution of asphalt after aging showed a shoulder peak in the range of 10^3.5^–10^4.5^. The relative content increased, which indicated that the aging of asphalt promoted the increase of the macromolecular content. At the same time, the macromolecular content of asphalt decreased after the addition of the regeneration agent. The shoulder in the range of 10^3.5^–10^4.5^ slowly became slower with the increase of the regeneration agent dose. The correlation study concluded a correlation between the molecular weight size of asphalt and asphalt properties. The more the macromolecular size in asphalt, the worse the asphalt performance [23]. The results of Jenning [23] and Kim [24] averaged the GPC data into 13 intervals with a sizeable molecular size (LMSs; intervals 1–5), a medium molecular size (MMSs; intervals 6–9), and a small molecular size (SMSs; intervals 10–13). Figure 11 shows the molecular size distributions of the asphalt specimens. As seen in Figure 12, the increase in the rejuvenation agent admixture and the regenerated asphalt exhibited higher MMSs and reduced LMSs. This is consistent with the GPC results, where adding a rejuvenation agent resulted in lower *M*_w_ and PD values. The LMSs of asphalt showed a better correlation with the asphalt properties than other dimensions [23]. Therefore, the study on the regeneration agent effect focused on LMSs, showing that LMSs significantly decreased when the regeneration agent dose was 6%, which coincided with the lower *M*_w_ and PD values found in the GPC test. It indicated that the regenerating agent could significantly reduce the asphalt macromolecular size. Although rejuvenation agents could replenish the components lost by asphalt due to aging, it was not easy to rebalance the distribution of regulated molecules. The GPC test results showed that the rejuvenator could reduce the size of large and medium molecules and increase the size of small molecules; the rejuvenator had the effect of decomposing the large and medium molecules in asphalt. At the same time, the proportion of heavy components in the asphalt was reduced, and the proportion of light components was increased after the incorporation of regenerants. This inevitably weakened the high temperature performance and the Brinell viscosity of the aged asphalt. However, whether the ratio of each component in the recycled asphalt is the same as that of the original asphalt needs further study and analysis.

### 3.4. FTIR Analysis

**Figure 13:**
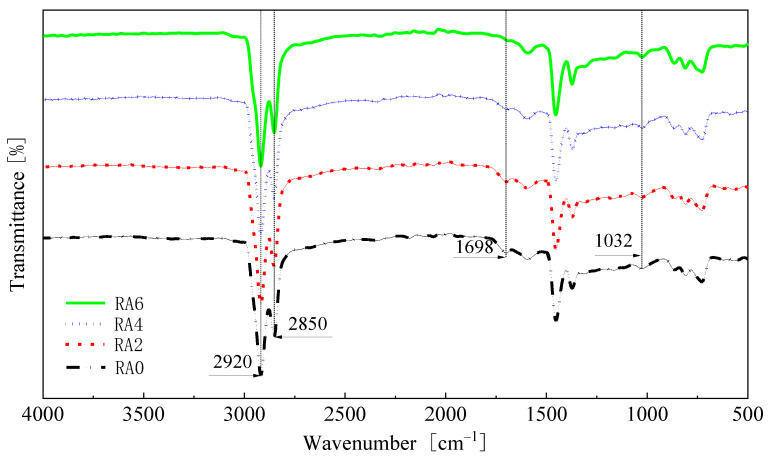
Infrared spectra of the asphalts.

**Figure 14:**
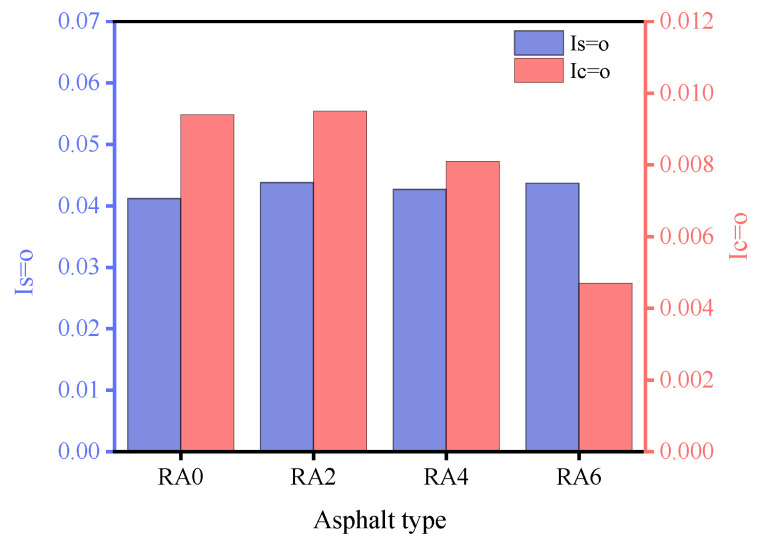
Sulfinyl and carbonyl index diagrams.

Figure 13 shows the infrared spectra of the asphalts in the wavenumber range of 4000 cm^−1^ to 500 cm^−1^. As shown from Figure 13, the infrared spectrum of the aged asphalt after the addition of the regenerating agent remained the same as that of the aged asphalt, and no apparent new absorption peaks appeared; the difference lied in the change of the transmittance size. The spectral difference between the aged asphalt and the regenerated asphalt was related to the functional group index. The functional groups that changed in the IR spectra were located at 1700 cm^−1^ and 1032 cm^−1^, representing the absorption peaks of carbonyl (C=O) and sulfoxide (S=O), respectively. Figure 14 shows the carbonyl and sulfoxide group indices for each asphalt specimen. We can see from Figure 14 that the sulfoxide index did not change significantly when the regeneration agent dose increased. When the regeneration agent dose was 2%, the carbonyl index also did not change. However, when the regeneration agent dose was 6%, the carbonyl index of the regenerated asphalt decreased by 50% compared with the aged asphalt, and the carbonyl functional group transmission rate of the regenerated asphalt increased significantly. The index of the carbonyl group showed a decreasing trend, when the doses of rejuvenator were 4% and 6%; it showed that the dose of rejuvenator that was greater than the dose of 2% was beneficial to reduce the content of heavy components in asphalts, so as to achieve the purpose of reducing the viscosity of the aged asphalt.

### 3.5. AFM Analysis

**Figure 15:**
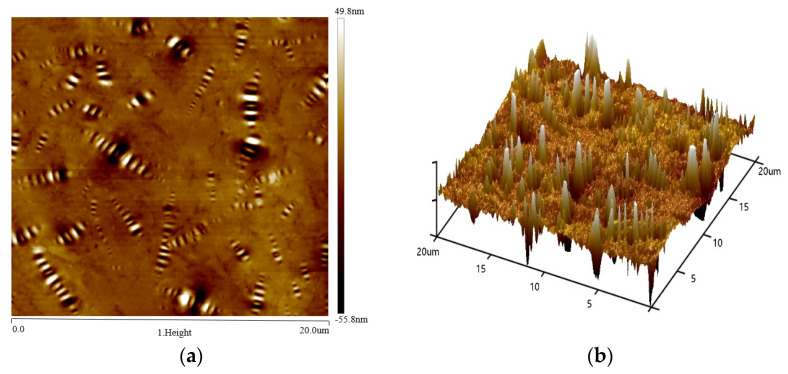
Two-dimensional (**a**) and three-dimensional (**b**) maps of the substrate asphalt morphology.

**Figure 16:**
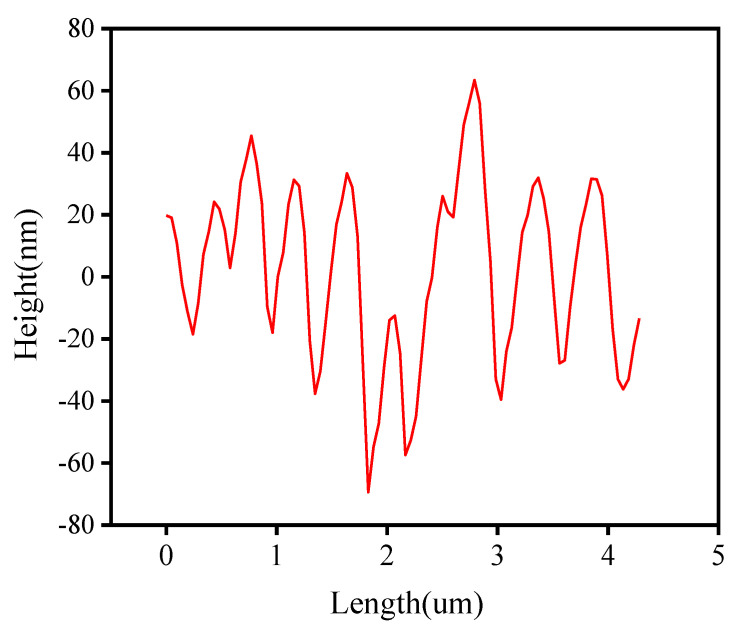
Bee-type structure cross-section.

Figure 15 shows the two-dimensional and three-dimensional morphology maps of the matrix asphalt. As seen in Figure 15, the microscopic surface of the asphalt was widely distributed with a bee-like structure, which was called a “bee-like structure” in the current study [25]. The colors in the asphalt morphology diagram only represent the height of the microscopic morphology and do not represent the actual color of the asphalt sample. A bee-like structure in the matrix asphalt was randomly selected using NanoScope analysis software to extract its height profile information. As shown in Figure 16, the height of this bee-like structure had a “wavy” shape along the horizontal axis. The peaks correspond to lighter areas of the morphology, while the valleys correspond to the darker areas of the morphology. Numerous studies have shown that the bee-like structures on the microscopic surface of asphalt are wax crystals formed by the eutectic of wax molecules and long-chain alkyl groups of polar macromolecules, and the molecular motility of asphalt significantly affects its aggregation state [26,27,28].

**Figure 17:**
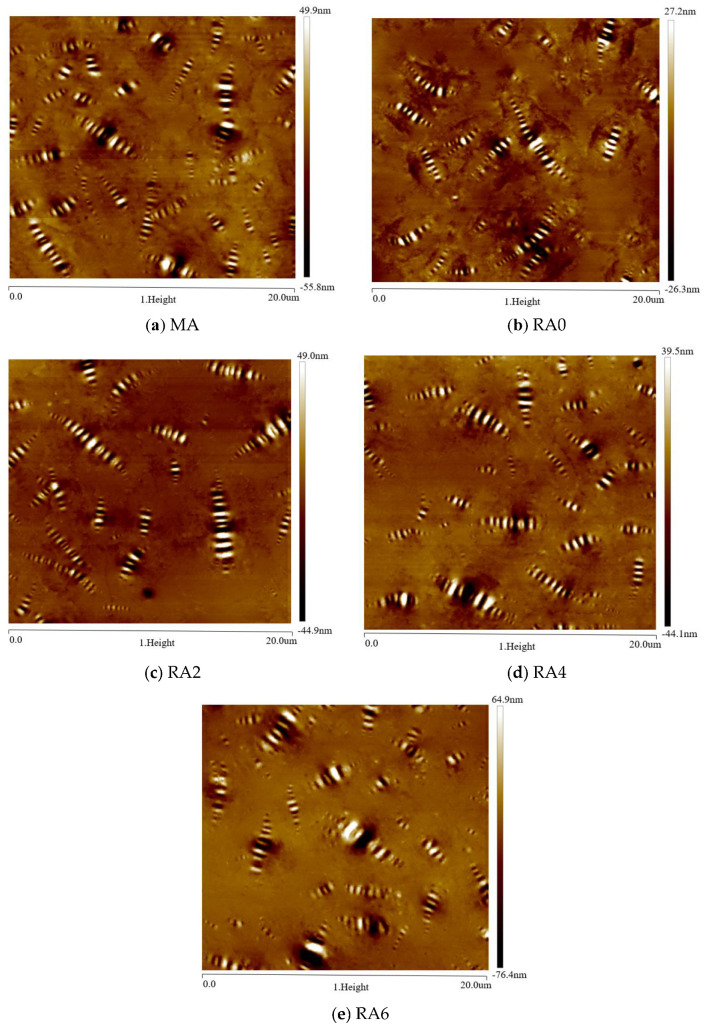
AFM morphologies of different asphalts.

Figure 17 shows the 2D morphologies of the different asphalts. We can see that all asphalts showed a bee-like structure. There was no essential difference between the microstructures of the matrix asphalt, aged asphalt, and regenerated asphalt; the main difference was the size and number of the bee-like structures. The aged asphalt had a smaller number of bee-like structures and a larger size compared to the matrix asphalt. The addition of the regenerating agent increased the number of bee-like structures and decreased the size of bee-like structures, which was an improvement compared to those of the aged asphalt. The presence of macromolecules was the main reason for the formation of bee-like structures. The addition of regenerating agent regulated the asphalt components and reduced the content of macromolecules in the aged asphalt, which is consistent with the results of GPC analysis. Table 5 and Figure 16 show the number of bee-like structures, the area of individual bee-like structures, and the roughness parameters.

**Figure 18:**
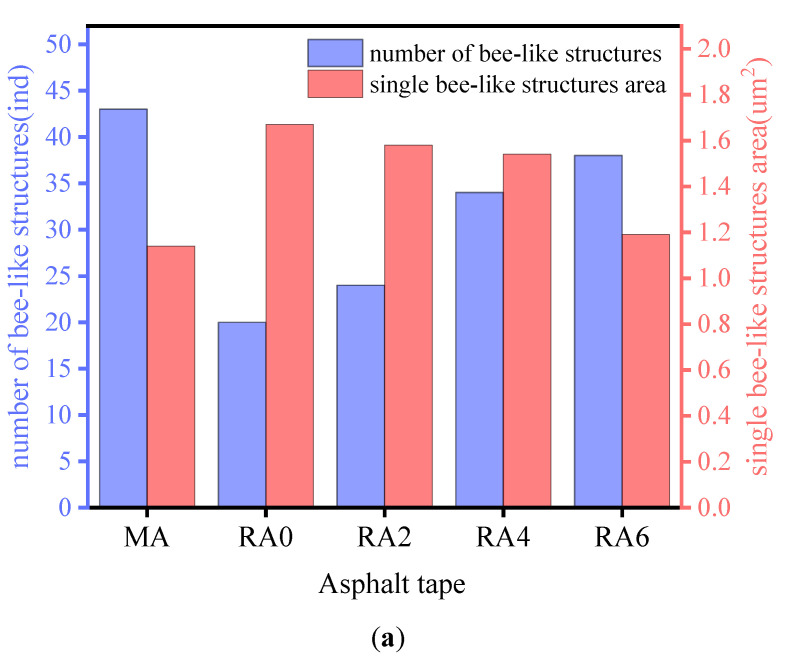

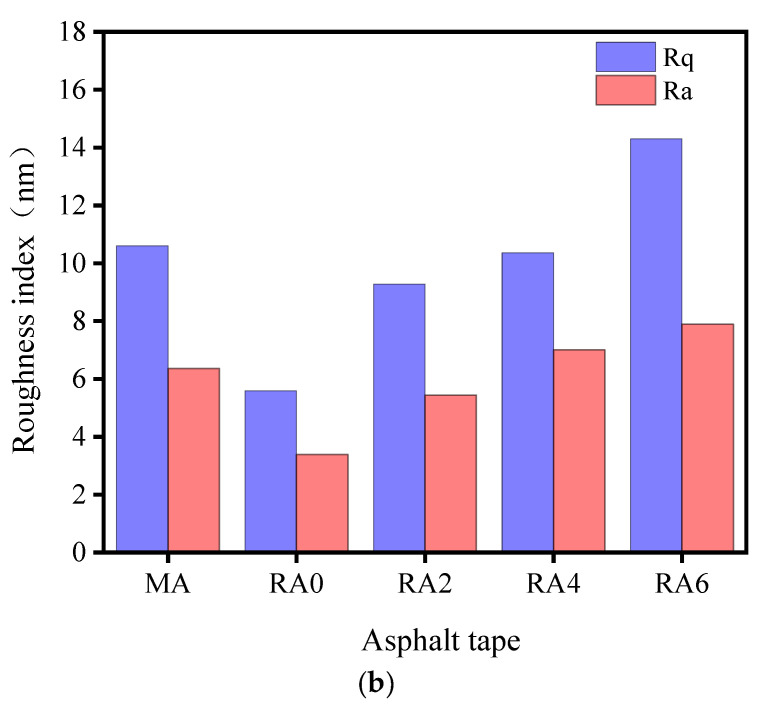
Variations of different asphalt parameters. (**a**) Trends of different asphalt bee-like structure indicators. (**b**) Trends of different asphalt roughness indices.

As seen in Figure 18, the number of bee-like structures of the matrix asphalt decreased by 50% after short-term aging. The number of bee-like structures increased with the dose of the revertant. The aging process increased the area of the individual bee-like structures of the matrix asphalt. The area of the individual bee-like structures of the asphalt decreased after the addition of the rejuvenation agent. The more the dose of the rejuvenation agent, the more the area of the individual bee-like structures of the asphalt decreased. The asphalt surface roughness *R_q_* and *R_a_* increased gradually with the increase of the rejuvenation agent dose. The results showed that aging led to the increased asphaltene content, providing a crystalline core for wax molecules, which helped reduce the number of bee-like structures and increase their area. The addition of a regenerating agent had a degrading and diluting effect on the internal macromolecules of the asphalt, thus reducing the viscosity of the asphalt. Consistent with the results of the GPC and FTIR tests described above, the rejuvenator helped to reduce the proportion of heavy components in the aged asphalt and to convert the heavy components to light components by physical or chemical action, thus reducing the asphalt viscosity and softening the aged asphalt. The microscopic level analysis of the rejuvenation agent can improve the construction and ease of the aged asphalt.

## 4. Conclusions

The significance of this study was to analyze the effect of the recycler dosing on the high-temperature performance and viscosity of recycled asphalt at the macroscopic and microscopic levels, to provide a scientific basis for the dosing of the recycled asphalt pavement, the mixing temperature of the recycled asphalt mixture, and the recycler dosing, to maximize the reuse rate of recycled asphalt pavement and to optimize the overall performance of the recycled asphalt mixture. In this paper, the changes of the relative molecular weight, the chemical composition, the microstructure, and the viscosity properties of asphalt before and after regeneration were analyzed by GPC, FTIR, AFM tests, and the asphalt activation energy. This paper also explored the microscopic change mechanism of the regenerated asphalt. The following main conclusions were obtained in this paper.

1. The addition of a regenerating agent significantly improved the flexibility and viscosity of aged asphalt but harmed the softening point. The regenerating agent could effectively reduce the viscosity of the aged asphalt, positively improving the construction and ease of the aging asphalt.
2. The FTIR results showed that the regenerating agent did not chemically react with the asphalt to produce new substances and the regenerating agent with a dose of 6% could significantly adjust the ratio of asphalt components. The regenerating agent could affect the contents of the sulfoxide group and carbon group in the regeneration process of aging asphalt and reduce the strength of its functional group index.
3. The GPC and AFM results showed that the content of macromolecules and the area of individual bee-like structures decreased with the rejuvenation agent dose. In addition, the number of bee-like structures on the microscopic surface of asphalt increased with the rejuvenation agent. We can also see that the rejuvenation agent had a degrading and diluting effect on the aging asphalt macromolecules, leading to a decreased asphalt viscosity.

## Figures and Tables

**Table 1:** Basic properties of 70# base asphalt.

Projects	Test Values
Penetration (25 °C, 0.1 mm)	60.4
Softening point (°C)	49.7
Ductility (15 °C, cm)	>100
Viscosity at 135 °C (Pa·s)	0.475

**Table 2:** Basic properties of the regeneration agent.

Physical Form	Density at 25 °C (g·cm^−3^)	Specific Gravity	Flash Point (°C)
Brown liquid	0.946	0.95	209

**Table 3:** Sample descriptions.

Specimen Number	Aged Asphalt Ratio (%)	Rejuvenation Agent Content (%)	Remarks
MA	0	0	Matrix asphalt
RA0	100	0	Aging asphalt
RA2	100	2	Regenerated asphalt
RA4	100	4	Regenerated asphalt
RA6	100	6	Regenerated asphalt

**Table 4:** GPC parameters of different asphalt specimens.

Asphalt Type	*M*_n_ (g·mol^−1^)	*M*_w_ (g·mol^−1^)	PD
MA	593	1600	2.69
RA0	595	1895	3.18
RA2	569	2009	3.53
RA4	563	2060	3.65
RA6	522	1694	3.24

**Table 5:** Results of parameters for different asphalts.

Parameters	MA	RA0	RA2	RA4	RA6
N (number)	43	21	27	35	38
Single area (μm^2^)	1.14	1.57	1.40	1.39	1.18
*R_q_* (nm)	10.60	5.59	9.28	10.36	14.30
*R_a_* (nm)	6.36	3.39	5.44	7.00	7.89

## Data Availability

The experimental and modeling data used to support the findings of this study are available from the corresponding author upon request.
